# Mycosynthesis of selenium nanoparticles using *Penicillium tardochrysogenum* as a therapeutic agent and their combination with infrared irradiation against Ehrlich carcinoma

**DOI:** 10.1038/s41598-024-52982-9

**Published:** 2024-01-31

**Authors:** Abeer I. M. EL-Sayed, Mostafa M. El-Sheekh, Sahar E. Abo-Neima

**Affiliations:** 1 Botany and Microbiology Department, Faculty of Science, Damanhour University, Damanhour, 22511 Egypt; 2 Botany Department, Faculty of Science, Tanta University, Tanta, 31527 Egypt; 3 Physics Department, Faculty of Science, Damanhour University, Damanhour, 22511 Egypt

**Keywords:** Biophysics, Drug discovery, Microbiology

## Abstract

Over the past years, the assessment of myco-fabricated selenium nanoparticles (SeNPs) properties, is still in its infancy. Herein, we have highly stable myco-synthesized SeNPs using molecularly identified soil-isolated fungus; *Penicillium tardochrysogenum* OR059437; (PeSeNPs) were clarified via TEM, EDX, UV–Vis spectrophotometer, FTIR and zeta potential. The therapeutic efficacy profile will be determined, these crystalline PeSeNPs were examined for antioxidant, antimicrobial, MIC, and anticancer potentials, indicating that, PeSeNPs have antioxidant activity of (IC_50_, 109.11 μg/mL) using DPPH free radical scavenging assay. Also, PeSeNPs possess antimicrobial potential against *Penicillium italicum* RCMB 001,018 (1) IMI 193,019, *Methicillin-Resistant Staphylococcus aureus* (MRSA) ATCC 4330 and *Porphyromonas gingivalis* RCMB 022,001 (1) EMCC 1699; with I.Z. diameters and MIC; 16 ± 0.5 mm and MIC 500 µg/ml, 11.9 ± 0.6 mm, 500 µg/ml and 15.9±0.6 mm, 1000 µg/ml, respectively. Additionally, TEM micrographs were taken for *P. italicum* treated with PeSeNPs, demonstrating the destruction of hyphal membrane and internal organelles integrity, pores formation, and cell death. PeSeNP alone in vivo and combined with a near-infrared physiotherapy lamp with an energy intensity of 140 mW/cm^2^ showed a strong therapeutic effect against cancer cells. Thus, PeSeNPs represent anticancer agents and a suitable photothermal option for treating different kinds of cancer cells with lower toxicity and higher efficiency than normal cells. The combination therapy showed a very large and significant reduction in tumor volume, the tumor cells showed large necrosis, shrank, and disappeared. There was also improvement in liver ultrastructure, liver enzymes, and histology, as well as renal function, urea, and creatinine.

## Introduction

Nanotechnology has made fast strides in several areas and applied in a wide range of vital industrial and medical sectors^1–3^. Nanotechnology has been a part of daily life during the past few decades thanks to a variety of uses^4^. This enhanced the opportunities for the creation of novel methods that improve efficacy, cytotoxicity, environmental consciousness, and the biohazardousness of nanoparticles formed because conventional operations applied in industry call for difficult systems and use elevated pressures and temperatures, resulting in potential ecological risks^5^. Additionally, nowadays, new ideas appear to push for the creation of a novel alternative nanotechnology technique that encourages the synthesis of nanomaterials utilizing biological processes like those used by plants or bacteria^6^.

Based on microorganisms' ability to transform metal ions into their nano-sized structures, microbial nanotechnology is a growing study field^7^. Two intracellular and extracellular routes are used in the microbial production of NPs^8^. The intracellular pathway creates NPs within cells, and additional downstream mechanisms are needed to liberate the nanoparticles. Finding the microbes that could create NPs extracellularly is therefore interesting. It has been widely reported that fungi are used in the extracellular production of NPs^8^. However, some fungi can spread illness^7^. Screening harmless fungi for NP production could therefore assist in the creation of medicinal applications for these NPs.

The interesting microorganisms are fungi, which are included in the environmentally friendly biosynthesized nanoparticles^9^. Many fungal categories could be great candidates to form a variety of NPs due to their adaptability, significant metal patience, simplicity in handling, high biomass output, and economic feasibility^10^. Because of their elevated scalability, manageability, and minimum price, the myco-produced NPs are denoted as "biofactory". The anticancer, antibacterial, and antioxidizing properties of selenium NPs prepared from living organisms make them a prospective resource in modern medicine^7,11,12^. Among the well-known fungi, *Penicillium chrysogenum*, offers several metabolites, such as chrysogine, hydroxyemodin, and chrysogenin, as well as different, roquefortines, penitric acid, enzymes, siderophores, and indole-3-acetic acid^13^. As a result, a range of metal oxide and metal nanoparticles might be created using it.

The basic chemical element that is widely spread in the environment is selenium (Se). It is also found in the crust of the earth in both organic and inorganic redox states, a strong antioxidant that inhibits cancer from starting, spreading, and growing while having no negative side effects. There are numerous additional positive effects on human health as well. However, Se and its states are home to a diverse range of biological activities and availability. In biomedicine, SeNPs are frequently used because of their wide range of biological activities and elevated bio-accessibility. They engage in a variety of biological processes, including anticancer and antibacterial ones. When compared to the commercial medication ampicillin, they exhibit promising antibacterial action against *Staphylococcus aureus*^14–18^. The National Academy of Sciences advised individuals to consume 55g of selenium each day^19^. Se NPs also demonstrated protection against cardiovascular disorders^7^ as well as alcohol-induced oxidative challenge^20^. The potential of SeNPs to boost selenoenzymes such glutathione peroxidases was comparable to that of other selenium supplements like L-selenomethionine^7^.

We created the present study to assess the capability of our Egyptian soil isolate *P. tardochrysogenum* OR059437 for SeNPs biosynthesis, in light of the promising benefits of biogenic SeNPs and their urgent use for the identification of ecologic and microorganisms which are non-pathogenic with excellent effectiveness for SeNP preparation in the field of microbial nanotechnology. Additionally, SeNPs a well-known, significant source of several enzymes with numerous uses in biotechnology and the creation of nanomaterials^21^.

Trace elements including selenium is essential for people, animal, and microbial wellness. It is also a dietary supplement^22–24^. Mineral deficiencies are associated with high mortality from cancer, infectious diseases, and cardiovascular diseases^25–27^.

Cancer is one of the diseases in which cells divide randomly. For several years, efforts have focused on identifying cancer risk factors. An unhealthy lifestyle such as smoking, stress, an imbalanced diet and insufficient exercise have a major impact on the risk of cancer^28,29^. Surgery, chemotherapy, radiation therapy, and hyperthermia are different therapeutic methods for treating cancer^30,31^.

Chemotherapy, a common treatment for unresectable cancer, often causes serious side effects as the drug enters normal cells^32^. Due to this negative impact, people cannot tolerate the elevated dosages of chemo-treatment needed to eradicate tumors. Additionally, most cancer cells eventually develop drug resistance, leading to death. Therefore, the development of new drugs or innovative treatment techniques is necessary. According to Salama et al.^33^. Radiotherapy is a highly successful and targeted treatment for some tumors with minimal metastases. This procedure utilizes radiation with high energies, such as particles with charges, rays, and X-rays. According to^34^ radiation with high energies causes DNA double-strand splits, which promote the death of tumor cells.

One form of cancer treatment is combination therapy with two types of treatment, such as hyperthermia with radiation therapy. Combination therapy is a more effective treatment and reduces the toxic effects of chemotherapy. The results are much better than monotherapy. Since cancer stem cells are known to survive after monotherapy and can therefore traverse blood arteries and become active again, combining medications helps eliminate tumor stem cells, which are implicated in resistance to therapies and malignancy recurrence in the years following recovery^35,36^.

Ultimately, we try to achieve the maximum effectiveness with the least toxicity by using two different methods as a combination therapy to fight cancer. It's critical to comprehend the interaction between the two methods to get better results. The immune system is impacted by heat, and when a tumor is locally hot, the body's defense system is activated and a reaction of immunity takes place.

Treatment with hyperthermia by raising the temperature of the tumor tissue with a group of nanomaterial that accumulates in the tissue and becomes a heat source by activating Nano selenium with infrared light. Heat treatment must be safe and effective without causing excruciating burns, swelling, and bleeding. Hyperthermia is a successful adjuvant therapy that delivers nanoparticles and specifically targets the spots of tumor^37^.

Despite being used as an additive, hyperthermia causes direct cell destruction by damaging lipoprotein cells and also leads to the disassembly and denaturation of intracellular proteins and the regulation of cytoskeletal proteins^38^. It involves heating the tumor site by introducing SeNPs into the tumor area and then irradiating the tumor area with infrared light, the infrared rays being absorbed in the SeNPs samples to heat the SeNPs contained therein and thus achieve better treatment results^39^.

The current work aims to test our myco-synthesized PeSeNPs in vitro against harmful pathogens by studying the antioxidant, and antiviral potentials, and in vivo study against tumor cells combined with infrared radio-medication for studying efficacy of combined therapy against Ehrlich ascites carcinoma cells.

## Materials and methods

### Isolation and molecular identification of fungus

The chosen isolated fungus was found among soil samples taken from Alexandria, Egypt; samples were taken from three to four centimeters deep by a clean sterile spatula. They were then transferred to the microbiology lab to be preserved for further study after being put in sterile plastic bags. Using Sabouraud dextrose agar medium (pH 5.5), the conventional serial dilution procedure was used to isolate the fungus from 10^− 1^ to 10^− 5^ on Sabouraud dextrose agar medium (pH 5.5), isolation was carried out, and it was cultured in a static condition at 28 °C for five days. After the media had cooled, 1000 µl per liter of streptomycin were added^40^.

DNA sequence analysis was done by using a large ribosomal subunit, incomplete sequence 28S rRNA by universal primers for fungi, and gene sequencing analysis. Sigma Scientific Services Co., Giza, Egypt (http://sigmaeg-co.com/) experimented, *COSMO* PCR RED Master Mix (W10203002) was used to amplify the extracted DNA, which was extracted using the Quick-DNA Fungal/Bacterial Mini-prep Kit (Zymo Research #D6005).

The PCR program was performed by the following cycling parameters: Initial denaturing was done for 3 min at 95 °C. 35 cycles were denatured for 30 s. Annealing was done for 30 s. At 52 °C, the annealing was extended for 2 min. At 72 °C, the final extension was completed for 10 min.

An accession number was assigned, and the data were entered into the National Center for Biotechnology Information (https://blast.ncbi.nlm.nih.gov). The phylogenetic tree was done using MEGA11.

### Biosynthesis of PeSeNPs

The fungus isolate was cultured in a 250 ml conical flask at 28 °C for five days while being in a static incubator with 100 ml of sterile Sabouraud dextrose broth medium at pH 5.5. To separate the fungal biomass from the culture broth, Whatman filter paper no. 1 (Whatman, England) was utilized after the complete incubation period. This process involved washing the fungal biomass 3times with sterile double-distilled water to get rid of any impurities from the medium. Then, the fungus's biomass was eliminated and placed in an Erlenmeyer flask (250 ml) with 100 ml of distilled sterile water and shaken for 3 days at 28 °C at 150 rpm. By using Whatman No. 1 filter paper, it was filtered after the incubation period. Cell-free filtrate (CFF) must be obtained^41^. The Na_2_SeO_4_ was supplied by Sigma-Aldrich, Inc., St. Louis, Missouri, USA, in a water solution of concentration 1 mM. After adding 100 ml of CFF to the mixture, it was shaken for 72 h at 150 rpm at 28 °C in the dark. Along with the test flasks, a control (without Se ions) was also prepared^42^. The produced extracellular Se-NPs were centrifuged for separation at 10.000 rpm for 30 min. following incubation time, and they were then employed in more investigations. The distinctive darkening of the mixture's color to red served as proof that Se-NPs had been successfully synthesized.

### Techniques used for characterization of PeSeNPs

#### Optical properties: UV–Vis spectroscopy

The absorption spectrum of Mycosynthesized selenium nanoparticles using *P. tardochrysogenum* OR059437 was obtained using a UV–Vis—NIR Australia Spectrophotometer. For this analysis 3ml of the filtrate sample was taken out of the flask at regular intervals for this investigation. A quartz cuvette containing the reaction mixture was used to measure the absorbance between 200 and 800nm. This graph was created by plotting absorption versus wavelength^43^. PeSeNPs were found. The generated nanoparticles were subsequently kept after drying at 50 °C for additional usage research.

#### TEM analysis of *PeSeNPs* sample

*PeSeNPs* size, shape, and morphology using TEM—JEM-2100 (JEOL Ltd., Tokyo, Japan) spectroscopy analysis was investigated for detecting the size of nanoparticles and their form. Five microliters of PeSeNPs were desiccated for 48 h after being deposited on the copper grid's surface. Afterwards, pictures with a resolution of 7000 × to 8000 × were taken while the PeSeNPs had been examined at 300 keV voltage^44^.

#### Fourier transform infrared (FTIR) spectroscopy

FTIR spectroscopy (FT/IR-6100 type A) was applied to identify the functional groups that acted as capping and stabilizing agents during the synthesis of PeSeNPs, which can provide reduction and capping for PeSeNPs in the region of 450–4000cm^−1^ wave number, The fungal extract's biomolecules' functional groups were determined. An IR spectrum obtained peaks is a plot of wavenumber, cm^−1^, (X-axis) versus percent transmittance or absorbance (Y-axis)^45^.

#### Zeta potential of the synthesized PeSeNPs

The zeta potential is an extremely important measure for determining the surface charge and behavior of colloids or nanoparticles in suspension. Its value correlates strongly with the shape of the particle surface and the stability of the suspension. Sonication was used to create a uniform suspension of nanoparticles, which was then centrifuged for 20 min at 6000 rpm. After that, a nanoanalyzer (Malvern 3000 Zetasizer Nano ZS, UK) was used to analyze it at 3.4eV and to evaluate the effective PeSeNPs surface charge and their long-term stability under numerous factors^46^.

### Energy dispersive X-ray analysis

The PeSeNPs were completely dry before being examined with an EDX between 0 and 12 keV. After that, a tiny section of the sample was covered with gold sputtering for 2 min while it was adhered to carbon tape. The elemental composition of PeSeNPs was then ascertained by analyzing it using the instrument (JEOL, JSM IT 500LA, Peabody, MA, USA)^47^.

### Antioxidant activity evaluation

PeSeNPs antioxidant activity was evaluated in triplicate at the Regional Center for Mycology and Biotechnology (RCMB) et al.-Azhar University in Cairo using the DPPH free radical scavenging method, with average data being considered. Radical Scavenging Activity in 2,2-diphenyl-1-picrylhydrazyl (DPPH): A fresh solution of methanol (0.004% w/v) was used to prepare the DPPH radical, which then remained at 10 °C in darkness. The test material was made into a methanol solvent. A 40 L aliquot of the ready-made methanol solution was mixed with the DPPH solution. To measure the instantaneous absorbance, a UV–visible spectrophotometer (Milton Roy, Spectronic 1201) was utilized. The decrease in absorbance at 515 nm was continuously observed, with data being recorded at 1 min intervals, till the absorbance was fixed (16 min). DPPH radical absorbance without an antioxidant (control) and ascorbic acid's absorbance were measured using the standard chemical as a guide. For every determination, there were three replicas, and the average was established. Equation (1) was used as the method for calculating the DPPH radical's percentage inhibition (PI).

1 $$ PI = \left[ {\left\{ {\left( {AC - AT} \right)/AC} \right\} \times 100} \right] $$

where *AC* = Absorbance of the control at t = 0 min and *AT* = absorbance of the sample + DPPH at t = 16 min^48^.

The 50% inhibitory concentration (IC_50_), or the concentration required to inhibit the DPPH radical by 50%, was derived from graphic representations of the dose–response curve.

### Antimicrobial activity evaluation

The Agar well diffusion technique^49^ was employed to measure the impact of our PeSeNPs, with gentamicin (4µg/ml) serving as an antibacterial antibiotic and ketoconazole (100μg/ml) serving as an antifungal antibiotic standard. Mueller Hinton Agar (MHA) medium for bacteria and Sabouraud Dextrose Agar (SDA) media for fungi were prepared for antifungal and antibacterial activity, sterilized, then spread evenly into Petri plates for maintenance of tested pathogenic fungal and bacterial strains, (*Penicillium expansum* RCMB 001001 (1) IMI 28,169, *Penicillium italicum* RCMB 001,018 (1) IMI 193,019, *Penicillium marneffeii* (RCMB 001,022), *Methicillin-Resistant Staphylococcus aureus* (MRSA) ATCC 4330 as a gram-positive bacterium and *Porphyromonas gingivalis* RCMB 022,001 (1) EMCC 1699 as a gram-negative bacterium). 100µL of pathogenic fungi culture broth (3 × 10^3^ CFU /mL) and (10^6^ CFU /mL) of bacterial selected strains were gently spread into sterilized petri plates by a sterile glass spreader, petri plates were loaded with the specific medium for fungi and bacteria separately, and wells with 6mm diameter each were made using a sterilized cork borer. One well was loaded with100 μl of ketoconazole (100μg/ml); if this petri plate was seeded by fungus, the second well was loaded with 100 μl gentamicin (4 µg/ml); if this plate was seeded by bacteria, a third well was loaded by PeSeNPs with a concentration of (1mg/ml). Petri plates were incubated for 5 days at 28 ± 2 °C for fungi and 24-48h at 37 ± 2 °C for bacteria. Then, they were examined for inhibition zones. The positive control was ketoconazole (100 μg/ml) and gentamicin (4 µg/ml) as antifungal and antibacterial antibiotics. The negative control was distilled water^47^. The clear ruler was used to measure the inhibition zones (mm). The experiment was run in triplicate, and the data were presented as mean and standard deviation (SD)^50^.

### Minimum inhibitory concentration

The MIC of PeSeNPs with standard antifungal antibiotic ketoconazole (100 μg/ml) and antibacterial antibiotic gentamicin (4 µg/ml) were investigated via a micro-dilution assay according to Ericsson and Sherris^51^. 100μL of PeSeNPs separately at Different concentrations (1000–0.5 µg/ml) were tested to determine the MIC. Sabouraud dextrose broth (SDB) and Mueller Hinton broth (MHB) tubes that were already inoculated with a standard volume size of 100µl fungal spore suspension (3 × 10^3^ CFU/mL) and inoculated with 100 µl of bacteria (1.5 × 10^8^ Colony Forming Unit (CFU) /mL). Tubes of SDB and MHB were inoculated with tested microorganisms (*Penicillium italicum* RCMB 001,018 (1) IMI 193,019, *Methicillin-Resistant Staphylococcus aureus* (MRSA) ATCC 4330 and *Porphyromonas gingivalis* RCMB 022,001 (1) EMCC 1699) used as controls. All fungal and bacterial tubes were incubated for 5 days at 28 °C and 24-48h at 37±2 °C, respectively. MIC readings were observed as the most minimal possible amounts from PeSeNPs preventing the visible growth of pathogenic microbes^52^.

### Transmission electron microscope

Using electron microscopy, modifications made in cells of pathogenic *Penicillium italicum* RCMB 001,018 (1) IMI 193,019 before and after MIC treatment of PeSeNps were demonstrated. Fungal cells from five-day cultures grown on SDB media were centrifuged for separation (at 4000 rpm for 10 min); distilled water was then used to clean the fungal cells, fixed afterwards in 3% glutaraldehyde, cleaned in phosphate buffer, and then post-fixed in potassium permanganate solution at room temperature for 5 min. Samples were dehydrated for 15 min in each ethanol dilution, with concentration varying from 10 to 90%, and then for 30 min in absolute ethanol. Following a graduated series of epoxy resin and acetone injections, the samples were eventually immersed in pure resin. Small pieces were gathered onto copper grids. Subsequently, sections were dyed twice: once with uranyl acetate and once with lead citrate. Using a JEOL-JEM 1010 transmission electron microscope, sections were examined at 70 kV at The Regional Center for Mycology and Biotechnology (RCMB), Al-Azhar University^53–55^.

### In vivo* studies*

#### Experimental animals

All experimental protocols and animal testing have been approved by the Scientific Research Ethics Committee of the Faculty of Science at Damanhour University and implemented by the Guide to the Care of Laboratory Animals. In this study, 50 male Swiss albino mice obtained from VACSERA, Giza, Egypt almost eight weeks old and weighing between 20-25 g, were used and they were kept at room temperature and 55% relative humidity and 12 h light Cycles and darkness while consuming healthy food and water at certain times. All mice were inoculated with 0.2 ml of Ehrlich ascites carcinoma then used mice tumor volume ranged from 0.7–1 cm^3^, all mice were randomly divided into 5 groups (n = 10 for each group) as follows: Group 1: (NTBM: non-tumor bearing mice), which given 0.5 ml saline solution orally per day and served as negative control group. Group 2: Tumor-bearing mice (TBM) that didn't receive any treatment and were used as a positive control group. Group 3: (Infrared) TBM exposed to infrared radiation therapy (140 mW/cm^2^) for 2 min for two weeks. Group 4: (PeSe-Nps) TBM receiving 225 µg PeSeNps/kg body weight for two weeks. Group 5: (Infrared + PeSe-NPs) TBM were injected with PeSe-NPs as in group 4, and then the tumor was exposed to radiation as in group 3.

#### In vivo* study*

##### Tumor volume

Solid tumors were generated by inoculating the right flank of mice with 0.2 ml EAC containing one million live EAC cells. Measurement of tumor volume over different periods 7,14,21,28 with calipers^42^.

##### Infrared light and Pe SeNPs therapy

*Exposure facility system*: An infrared physical therapy lamp for near-infrared light of 140 mW/cm^2^ is used. First, shave the hair covering the tumor. The mice were fixed on a board of board with the tumor elevated, and the infrared probe was firmly implanted and the tumor was exposed to infrared light for two min.

##### Tissue preparation

The mice were killed at the end of the experiment; all excited livers were divided into 3 parts. The 1^st^ part was used for a microscopic (Trinocular Biological Microscope 400x–600x, USA) histopathological analysis after being preserved in 10% formalin. The 2nd part was rinsed with 0.9% saline, then suspended in normal saline (0.5g tissue/5 ml saline) using a Teflon homogenizer used for homogenization. The homogenates were centrifuged, and the supernatant was stored at room temperature for further analysis. The 3rd part was fixed in glutaraldehyde and used for TEM studies^56^.

##### Biochemical assays

Renal function, involving urea and creatinine, and liver enzymes, such as aspartate aminotransferase (AST) and alanine aminotransferase (ALT), were measured in blood serum to determine liver and kidney enzymatic activities for all studied groups.

##### Histopathological examination

Livers, kidneys, and tumors were fixed in 10% formaldehyde buffered. and dehydration with ethanol in xylene solution for removable ethanol and facilitating wax melting of paraffin in the permeation process at 55°C. They are then dipped into a block of wax of 6μm thick paraffin sections cut with a rotary microtome and placed on cleaned glass slides. Finally, the sections were stained with eosin and hematoxylin. A light microscope was used to inspect the stained slides, and microscopic images of the tissue samples were captured^57,58^.

##### Transmission electron microscope examination

Liver samples were fixed in glutaraldehyde (2.5%) in cacodylate buffer, after which the tissue blocks were washed well in osmium tetroxide, followed by a graded series of ethanol dehydration (50%, 70%, 95%, and 100%) and purified in propylene oxide and then embedded in an araldite mixture. We utilized lead citrate and uranyl acetate to stain the ultrathin slices^59^.

### Statistical evaluation

All experiments were repeated 3times. The results were given as the mean value ± SE via the descriptive statistics frequencies with Statistical Package for the Microsoft Excel statistical program. ANOVA-test was used for statistical analysis. Our results were compared to determine the variation between the control and experimental groups.

### Ethics approval and consent to participate

All experimental designs were approved by the Research Ethical Committee, Faculty of Science, Damanhur University, Egypt by code number DMU-SCI-CSRE (23-10-01), following the international guidelines for animal care and the use of laboratory animals. The study is reported in accordance with ARRIVE guidelines.

### Informed consent

This work was not carried out on humans or animals.

## Results and discussion

### Molecular identification

**Figure 1:**
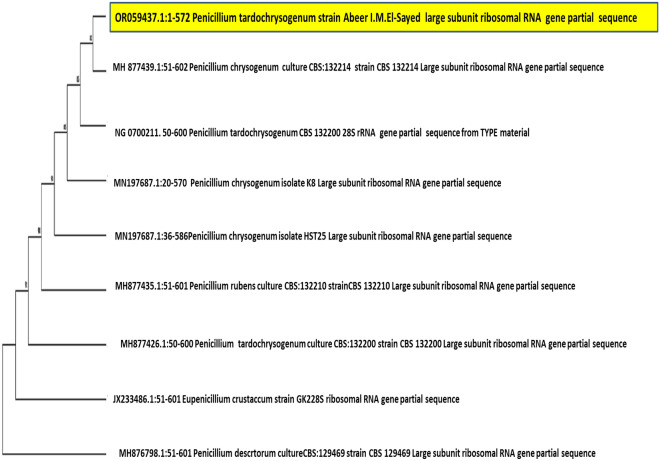
Neighbor-joining phylogenetic tree of *P. tardochrysogenum* OR059437and related fungi.

Our fungal isolate showed a high initial screening task and obvious growth; thus, it was also utilized for biosynthesizing our SeNPs and examining the antibacterial, antifungal, antioxidant and cancer-fighting properties^60^. Our isolated fungal species created white mycelia throughout its vegetative stage, and it developed green sporulation over the sporulation process^40^. The fungal isolate's 28S rRNA sequences were matched with genetically similar strains from GenBank. Under the accession number OR059437, the sequence was entered into the database of GenBank. A phylogenetic tree (Fig. 1) was established via the method of neighbor-joining in the MEGA11 software package to show the degree of similarity between the acquired sequence and the reference sequences in the genomic database.

### *SeNPs synthesis by P. tardochrysogenum* OR059437

**Figure 2:**
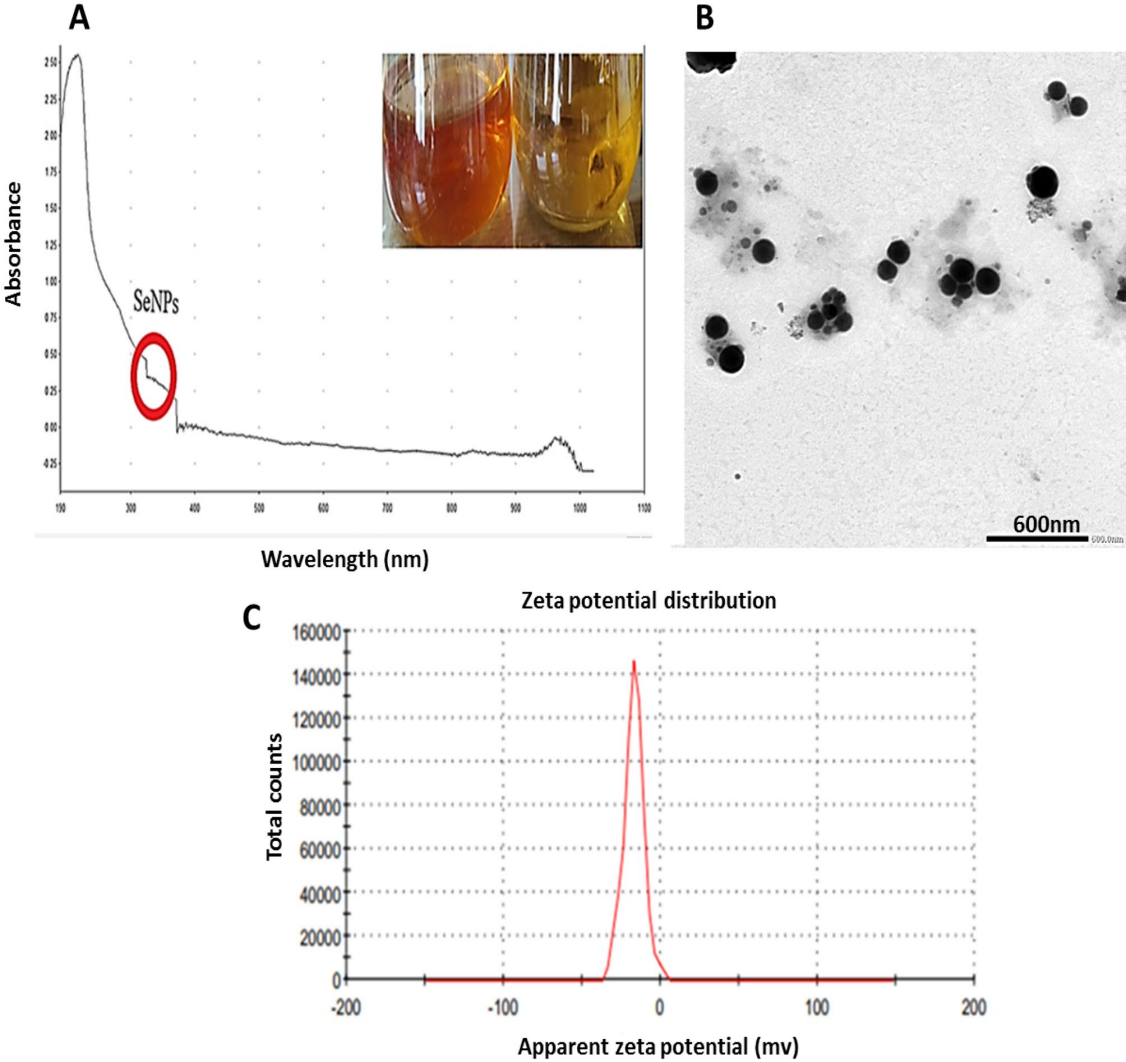
(**A**) UV- Visible spectrum of myco-synthesized Se-NPs using *P. tardochrysogenum* OR059437, (the red circle indicated the SeNPs formation range), (**B**): TEM image of myco-synthesized Se-NPs of scale 600 nm, (**C**): Zeta potential distribution of myco-synthesized Se-NPs.

Brick-red replaced the previous yellow hue in the culture medium after 30min of incubation when treating the culture filtrate with 1 mM Na_2_SeO_4_ (Fig. 2A).

The metabolites secreted extracellularly swiftly converted ions of selenite into elemental Se (Se0) form since culture media after incubation exhibited a red-brick appearance^61^.

### Characterization of Myco-synthesized SeNPs

Developing selenium NPs in the culture filtrate was observed by UV–visible spectrophotometry, which showed apronounced shoulder at about 350 nm, a SeNPs characteristic (Fig. 2A). This outcome is consistent with that of Yedurkar et al.^62^. The UV absorbance peak of the phycosynthesized *Polycladia myrica* SeNPs was 350. Experiments made by Morad, et al.^61^ illustrated a surface plasmon resonance (SPR) peak at 521 nm, that is a property of selenium NPs, was highly and broadly visible as a result of the formation of SeNPs in the filtrate of *Penicillium chrysogenum* Z945518.

A colloidal solution of myco-produced SeNPs was examined using TEM as shown in (Fig. 2B,C); indicating the production of poly-dispersed spherical SeNPs with diameters from 60.21 to 104.41 nm. These investigations are reliable with those made by El-Shanshoury et al.^63^, when examined *Bacillus subtilis* to create selenium nanoparticles and found that the resulting particles were polydispersed and spherical. The experiments made by Morad, et al.^61^, were acceptable with our findings as this previous study demonstrated SeNPs produced from *penicillium chrysogenum* Z945518, with sizes between 44 to 78 nm.

Depending on the zeta potential analysis of the charge on the particle's surface, the stability of selenium NPs was assessed, which resulted in a mean zeta potential of − 17 mV (Fig. 2D). Our measurements was acceptable based on the research of Morad et al.^61^, who claimed that the SeNPs formed by *penicillium chrysogenum* Z945518 observed a mean zeta potential of − 32.4 mV. Furthermore, according to Dumore and Mukhopadhyay^64^, it was found that the negative zeta potential is helpful for anti-oxidation and the use of nanoparticles in cancer (A549) cells. These observations may be attributed to the surface of SeNPs revealing the -ve charge of the OH and COO groups. The higher stability of these SeNPs is shown by a bigger zeta potential magnitude.

**Figure 3:**
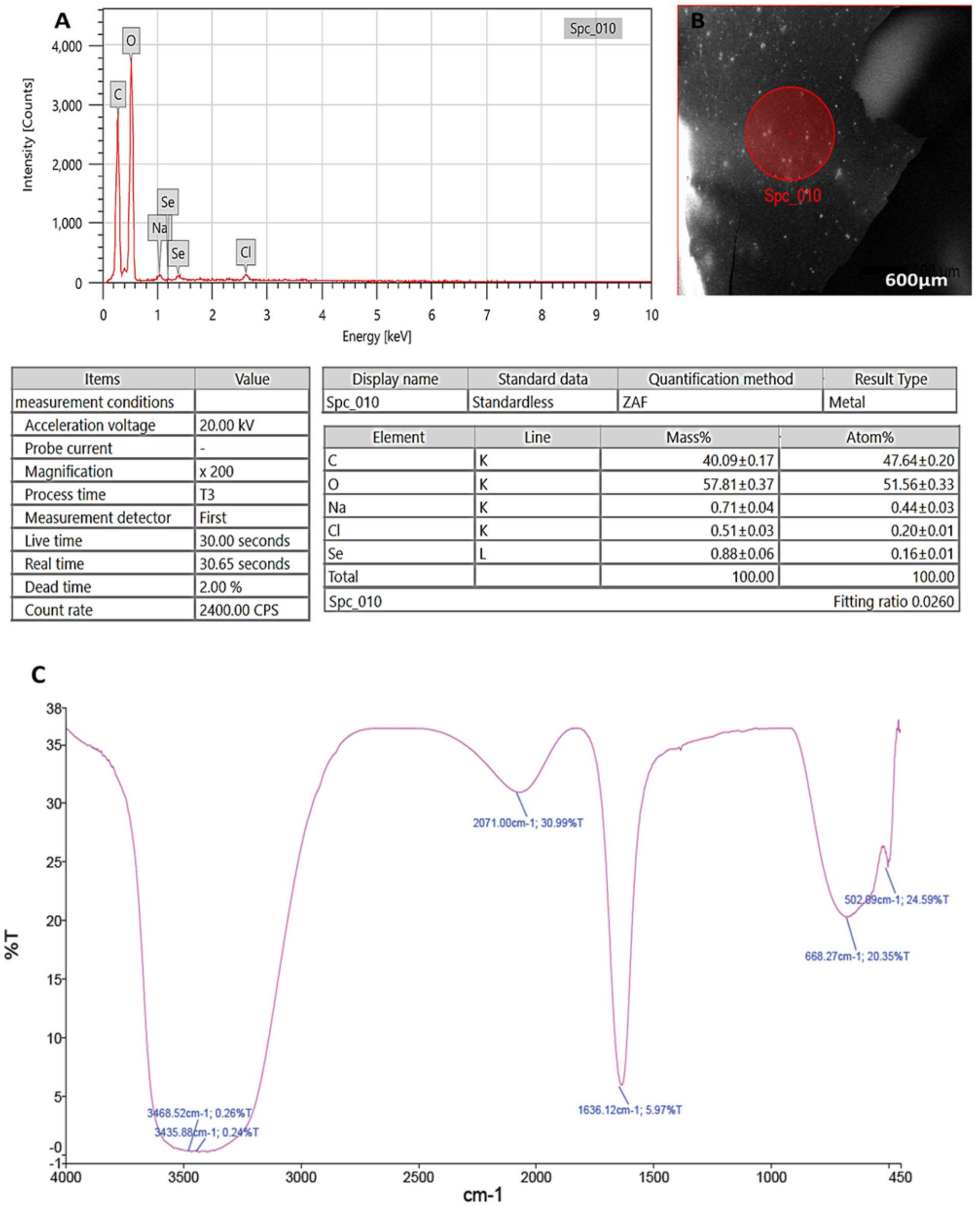
(**A**): EDX analysis of PeSeNPS, (**B**): Field of EDX analysis of PeSeNPs, (**C**): FT-IR spectrum of PeSeNPs.

Through EDX analysis; selenium elemental composition was shown in (Fig. 3A,B); PeSeNPs had an atomic and mass percentage: 0.16% ± 0.01 and 0.88% ± 0.06, respectively. This proves SeNPs were prepared. Some other noticed EDX peaks were recorded including C, O, Na, and Cl, with mass percentages of 40.09 ± 0.17, 57.81 ± 0.37, 0.71 ± 0.04, and 0.51 ± 0.03, atom percentages of 47.64 ± 0.20, 51.56 ± 0.33, 0.44 ± 0.03 and 0.20 ± 0.01, respectively.

FTIR analyses were utilized for identifying the appearance of several functional groups in metabolites that are in charge of SeNP synthesized from fungi, capping, and stability. The PeSeNPs' FT-IR spectra (Fig. 3C), exhibited absorption peaks at 3468.52, 3435.88, 2071.00, 1636.12, 668.27, and 502.09 cm^−1^ in the area of 450 to 4000 cm^−1^. The high absorption bands suggest the presence of alcohols at 3468.52–3435.88 cm^−1^ in the PeSeNPs illustrating the O–H stretching^65,66^. 2071.00 cm^−1^ band allocated to C=C stretching in alkenes, C=C phenyl compounds stretching, and C=O of aromatic amide I stretching (proteins and peptides) are represented by the band at 1636.12 cm^−1^ of the spectra, as stated by Demir et al.^67^, band at 668.27 cm^−1^ and 502.09 cm^−1^ bands were related to C–Cl stretching group.

In the same line Barabadi, et al.^68^**,** found that; *Penicillium chrysogenum* PTCC 5031; FT-IR spectrum SeNPs showed absorption peaks between 450 and 4000 cm^−1^ at 1088.38, 1412.66, 1632.57, and 3440.33 cm^−1^. The C–N stretching vibration of the amine is represented by the band at 1088.38 cm^−1^. Primary amides' N–H stretching vibration is represented by the band at 1412.66 cm^−1^. Additionally, C–C and O–H stretching were attributed to peaks at 1632.57 and 3440.33 cm^−1^, respectively. FT-IR results demonstrated the existence of functional groups on the surface of SeNPs. The observed functional groups are a result of the conjugated biomolecules acting as stabilizing and reducing agents on selenium nanoparticle’s surface.

### Antioxidant activity of myco-synthesized PeSeNPs

**Figure 4:**
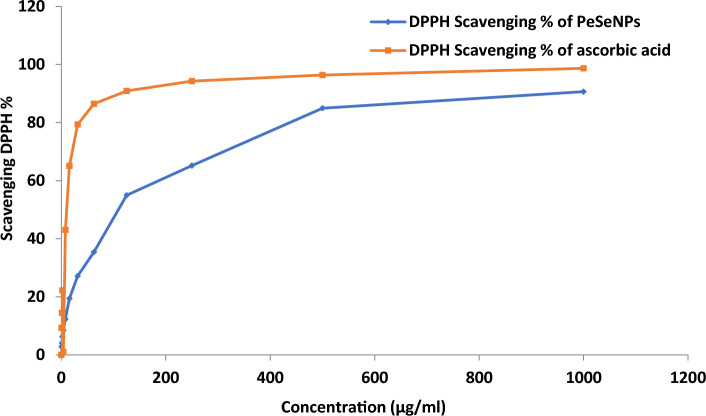
DPPH scavenging activity of PeSeNPs.

However, DPPH radical scavenging activity of PeSeNPs (Fig. 4) reached higher than 90% at 1000 µg/ml of PeSeNPs in this work without applying any stabilizer and capping agent. The findings showed that PeSeNPs have a stronger DPPH scavenging activity than other nanoparticles. Under these experimental circumstances, the PeSeNPs sample demonstrated antioxidant activity with an IC_50_ of 109.11 ± 3.62 µg/ml. DPPH scavenging activity of chitosan selenium (CS-SeNPs), carboxymethyl chitosan-selenium (CCS-SeNPs), and gum Arabic selenium nanocomposites (GA-SeNPs) of 0.6 mM reaches around 80%, according to several types of literature^69,70^. However, in this study, without the application of any stabilizers or capping agents, the DPPH radical scavenging activity of ASeNPs reached > 80% at 1ml ASeNPs. The findings showed that ASeNPs have a stronger DPPH scavenging activity than other nanoparticles.

### Antimicrobial activity of myco-synthesized PeSeNPs

PeSeNPs didn't have any remarkable antifungal potential on *Penicillium expansum* RCMB 001001 (1) IMI 28169 and *Penicillium marneffeii* (RCMB 001022). But a strong promising inhibitory action was recorded against *Penicillium italicum* RCMB 001018 (1) IMI 193019 and represented with diameters: 16 ± 0.5 mm, which was near to that done by ketoconazole antifungal antibiotic on the same genus; 18.2 ± 0.41 mm. However, a promising antibacterial effect was exerted by our myco-fabricated NPs on *Methicillin-Resistant Staphylococcus aureus* (MRSA) ATCC 4330 and *Porphyromonas gingivalis* RCMB 022001 (1) EMCC 1699; 11.9 ± 0.6 mm and 15.9 ± 0.6 mm, respectively. It was noticed that this inhibitory action exerted due to PeSeNPs was very near to that was done by the referenced antibiotics on the examined microbial pathogens. However, the 2 studied bacterial infections can be controlled in terms of growth by gentamicin; MRSA ATCC 4330, and *Porphyromonas gingivalis* RCMB 022001 (1) EMCC 1699, by inhibition clear zone; 15.0 ± 0.5 mm and 18.1 ± 0.5 mm, respectively, also, ketoconazole antibiotic can inhibit the growth of tested fungal pathogens; *Penicillium expansum* RCMB 001001 (1) IMI 28169, *Penicillium italicum* RCMB 001018 (1) IMI 193019 and *Penicillium marneffeii* (RCMB 001022) with obvious inhibition zones; 17.1 ± 0.5 mm, 18.2 ± 0.4 mm and 12.9 ± 0.6 mm, respectively, as shown in Table 1. Results were done in triplicate and mentioned as mean ± standard deviation. This was supported by the observations of Vahidi et al.^7^ who stated that the SeNPs myco-fabricated by *P. chrysogenum* PTCC 5031; exhibited antibacterial efficacy with zones of inhibition (ZOI) of 10 and 13 mm, respectively, towards gram-positive bacterial pathogens such *Staphylococcus aureus* and *Listeria monocytogenes*. In the same line with our results, the study done by Rudrappa, et al.^40^ mentioned that the silver nanoparticles prepared from *Plumeria alba* leaf (P-AgNPs) showed an antimicrobial potential depending on concentration, in which, higher inhibition was observed at 100 µg/mL P-AgNPs. The strongest antimicrobial potential was recorded towards (*S. pneumoniae* and *E. faecalis*) and *C. glabrata* fungus, on the other hand, the minimum antimicrobial potential was measured towards (*E. coli* and *E. aerogenes*). Another study done by Math, et al.^47^, mentioned that a high potential of antimicrobial was exerted by bio-fabricated silver nanoparticles using extract of *Cardamine hirsuta* (L.) leaf was noticed in *P. aeruginosa* by zone of inhibition reached approximately 16.8 ± 0.2, 20.5 ± 0.5, 22.3 ± 0.5 and 23.4 ± 1.0 mm at different concentrations prepared from the NPs prepared from the extract (25, 50, 75, and 100 µL). The lowest action measured on *S. aureus* with 11.5 ± 0.3, 13.3 ± 0.3, 14.6 ± 0.5, and 16.3 ± 1.0 mm zones of inhibition at various NPs volumes of (25, 50, 75, and 100 µL), respectively. A finding stated by Abbas et al.,^71^ mentioned that SeNPs with particle size ranging from 32.08 to 103.82 nm as showed by TEM micrographs fabricated from by *Fusarium semitectum*, could have promising antimicrobial potential against numerous bacterial pathogens such as *S. aureus*, *P. aeruginosa*, *A. baumanni,* and *K. pneumonia*.

**Table 1:** Antimicrobial activity of PeSeNPs.

Pathogenic strains	I.Z. of PeSeNPs (10mg/ml) (mm) ± SD	I.Z. of Gentamicin (4µg/ml) (mm) ± SD	I.Z. of Ketoconazole (100µg/ml) (mm) ± SD
*Penicillium expansum* RCMB 001001 (1) IMI 28,169	NA	–	17.1 ± 0.5
*Penicillium italicum* RCMB 001,018 (1) IMI 193,019	16 ± 0.5	–	18.2 ± 0.4
*Penicillium marneffeii* (RCMB 001,022)	NA	–	12.9 ± 0.6
*Methicillin-Resistant Staphylococcus aureus* (MRSA) ATCC 4330	11.9 ± 0.6	15.0 ± 0.5	–
*Porphyromonas gingivalis* RCMB 022,001 (1) EMCC 1699	15.9 ± 0.6	18.1 ± 0.5	–

*NA* no activity, *IZ* inhibition zone.

### Minimum inhibitory concentration of PeSeNPs

Minimum Inhibitory Concentration (MIC) of PeSeNPs on microbial-examined strains was detected. Promising inhibition was observed in the PeSeNPs concentration of 500*μg/ml* against *Penicillium italicum* RCMB 001018 (1) IMI 193019 and *Methicillin-Resistant Staphylococcus aureus* (MRSA) ATCC 4330; however, the growth was significantly inhibited at 1000μg/ml of PeSeNPs against *Porphyromonas gingivalis* RCMB 022001 (1) EMCC 1699 as illustrated in Table 2. The mycosynthesized PeSeNPs had superior antifungal activity against *Penicillium italicum* RCMB 001018 (1) IMI 193019 compared to other tested fungal strains. In accordance with our research, Gunti et al.^72^. manufactured SeNPs by an aqueous extract of *Emblica officinalis* fruit and recorded that SeNPs had more promising antibacterial activity towards the tested Gram-positive bacterial pathogens (*S. aureus* MTCC 96, *Enterococcus faecalis* MTCC 439 and *Listeria monocytogenes* MTCC 657) than other examined Gram-negative (*E. coli* MTCC 4), with (MIC) recorded of about 9.16 µg/mL, 16.17 µg/mL, 33.17 µg/mL and 59.83 µg/mL towards *S. aureus*, *E. faecalis*, *L. monocytogenes*, and *E. coli*, respectively. For example, Tran et al. ^73^. mentioned the preparation of Se-NPs stabilized by polyvinyl alcohol and observed the action of SeNPs that significantly inhibits growth of *S. aureus* at a concentration as low as 1µg/mL, however at all of the tested concentrations, no growth inhibition of *E. coli* was seen.

**Table 2:** MIC of PeSeNPs against pathogenic microbial strains.

Pathogenic strains	MIC of PeSeNPs (µg/ml)	MIC of Gentamicin (µg/ml)	MIC of Ketoconazole (µg/ml)
*Penicillium italicum* RCMB 001,018 (1) IMI 193,019	500	–	312.5
*Methicillin-Resistant Staphylococcus aureus* (MRSA) ATCC 4330	500	312.5	–
*Porphyromonas gingivalis* RCMB 022,001 (1) EMCC 1699	1000	625	–

### Transmission electron microscope

**Figure 5:**
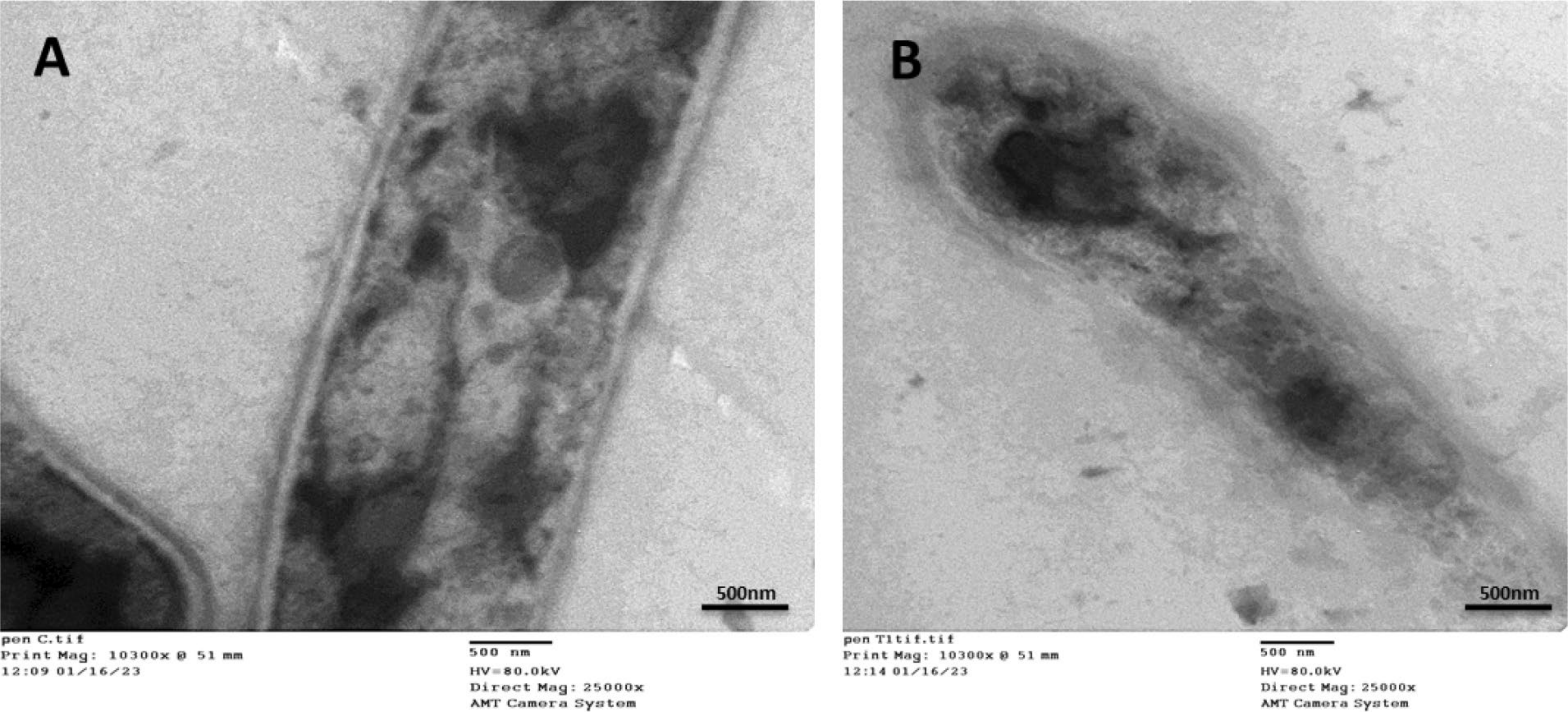
Transmission Electron microscopy images showing morphological alterations in *Penicillium italicum* RCMB 001,018 (1) IMI 193,019 hypha treated by myco-synthesized PeSeNPs. (**A**) showed the untreated *Penicillium italicum* RCMB 001,018 (1) IMI 193,019 exhibit a compact cell wall, continuous cytoplasmic membrane, homogeneous and electron-dense cytoplasm. (**B**) by contrast, the effect of treating *P. italicum* species with our myco- synthesized PeSeNPs; an obvious destruction and detachment of membrane and internal organelles integrity, subsequently, changes in hyphal shape formation of pores and cell death.

The TEM data offer a more comprehensive understanding of the cellular morphological deformations (Fig. 5A,B) showing the TEM micrographs of *Penicillium italicum* RCMB 001018 (1) IMI 193019 treated with PeSeNPs. The thin cross-section of individual *Penicillium italicum* RCMB 001018 (1) IMI 193019 cells is regarded as a control sample visible in micrographs Fig. 5A; as only cylindrical-shaped hyphae with rigid and obvious internal organelles. Once *Penicillium italicum* RCMB 001018 (1) IMI 193019 was treated with myco-synthesized PeSe-NPs; Fig. 5B, abnormal hyphal morphology with detached membrane and loosen appearance for the internal organelles observed, resulted in unusual impairment of hyphal membrane integrity. The fungal hyphal morphology showed a dramatic change from normal penicillium hyphae to a distorted structure with a broken weakened membrane.

Tran et al. and Zonaro et al.^73,74^; the following: size of the particle, shape, surface charge, surface chemistry, and hydrophilicity are significant factors that affect how microbial cell membranes are disrupted. SeNPs' potent electrostatic repulsion with bacterial membrane charge may be the cause of their greater antibacterial effectiveness towards gram-positive bacteria than gram-negative bacteria^71^. Tran et al.^72^, illustrated that Gram-positive bacteria have a significantly lower membrane-negative surface charge than Gram-negative bacteria, which makes it easier for Se-NPs to deposit on their surfaces and induce bacterial harm. As a result, Se-NPs are typically resistant to gram-negative bacteria. Guisbiers et al.^75^ noted that chemisorption is a potential method for accessing the Se-NPs in the bacterial cell. Lipopolysaccarides are found in the gram-negative bacteria's outer membrane, which Braun's lipoprotein uses to form a covalent bond with the peptidoglycan of the cell. Gram-positive bacteria's cell wall, which has a thicker peptidoglycan membrane, lacks the outer lipopolysaccharide membrane. Therefore, it would seem that SeNPs enter gram-positive bacteria through chemisorption considerably more readily. The selenium NPs disrupt both the wall and the membrane of the bacterial cell, penetrate the cell, frequently overproduce reactive oxygen species (ROS), interfere with ATP synthesis and respiratory sequence, cause protein denaturation, inhibit the activity of enzymes, cause DNA damage, and other effects that collectively cause the internal metabolism to fail consistently, leading to cell death^73^.

### Anticancer activity

#### In vitro* studies*

##### PeSeNPs cytotoxic activity

**Figure 6:**
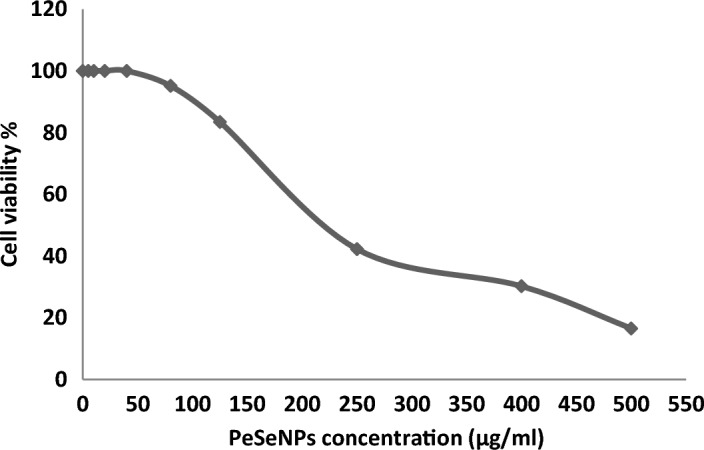
Effect of different doses of PeSeNPs on EAC cell viability assessed using the MTT assay, which indicates the cytotoxic activity against EAC cells with 50% cell cytotoxic concentration (IC_50_ = 225 ± 0.56 µg/ml).

Ehrlich ascites carcinoma EAC Cells were handled with various concentrations of Se-NPs 5, 10, 20, 40, 80, 125, 250, 400, and 500 µg/ml, and the cytotoxicity (Table 3, Fig. 6) transpired in ways necessary for its anticancer efficacy. All concentrations showed cytotoxic activity except (5, 10, 20, and 40 µg/ml), which showed 0% inhibition and 100% cell viability.

**Table 3:** Cytotoxic activity (n = 3) against EAC with 50% cell cytotoxic (IC_50_ = 225 ± 0.56 µg/ml).

Sample concentration (µg/ml)	Viability %	Inhibitory %
0	100	0
5	100	0
10	100	0
20	100	0
40	100	0
80	95.16	4.84 ± 0.32
125	83.46	16.54 ± 0.64
250	45.23	54.77 ± 0.72
400	30.22	69.78 ± 0.39
500	16.54	83.46 ± 0.54

The data are expressed in the form of mean ± standard deviation.

**Figure 7:**
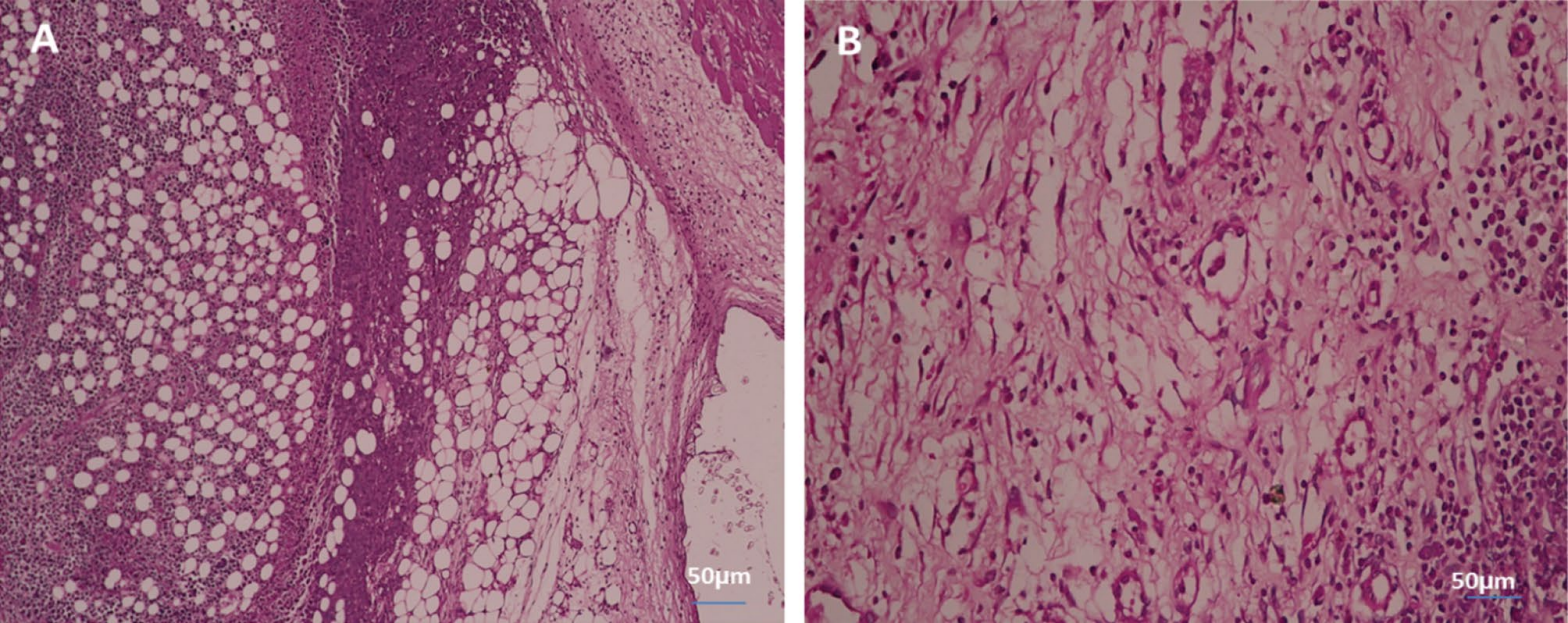
Cytotoxicity of PeSeNPS toward EAC. (**A**) The control EAC without PeSeNPS, and (**B**) EAC at a 225 µg/mL concentration.

**Figure 8:**
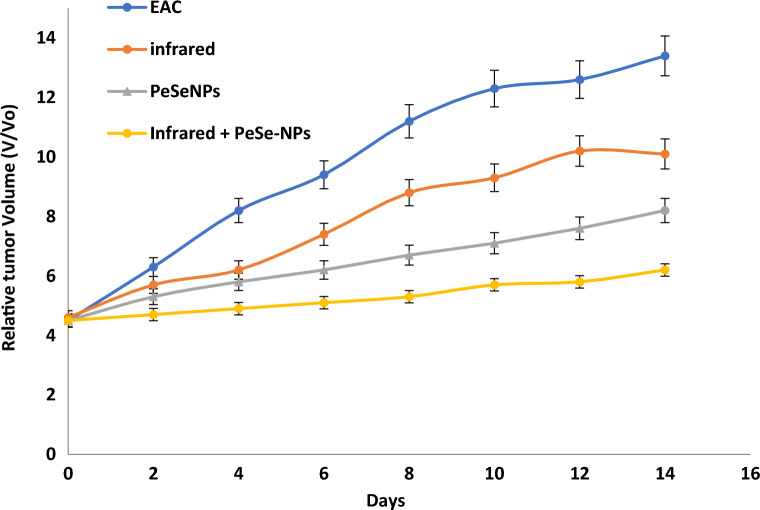
Effect of different treatments showing change in the relative tumor volume.

Kong et al.^76^ concluded that PeSeNPS inhibited Ehrlich ascites carcinoma (EAC) cell growth partially through caspases-mediated apoptosis and inhibited the transcriptional potential of the androgen receptor by downregulating its mRNA and expressing its protein. Apoptosis is a key mechanism for suppressing cancer growth, and caspases in turn cause apoptosis, as shown in (Fig. 8) by the difference between (Fig. 7A) of control cells without treatment at 100% and cell viability and (Fig. 7B) of cells treated with the maximum concentration of PeSeNPS (225 µg/ml) and cell viability 16.54%. Furthermore, selenium nanoparticles regulate Mdm2 degradation via the pathway of EAC tumor cell growth suppression by the breakage of androgen receptor, and phosphorylation promoting Akt-dependent androgen receptor, as well as increasing Akt kinase phosphorylation in order to be activated, act as a promising.

#### In vivo* studies*

##### Tumor volume

At the day 14, we start determining any change in tumor volume (Fig. 8). Tumor growth was consistent in control (EAC), with infrared radiation only, with PeSeNPs only, and by combining infrared radiation with PeSeNP groups. The relative tumor volumes (V/V_o_) on the 14th day are 13.4 ± 0.32 in EAC group, 10.1 ± 0.35 in the infrared radiation group, and 8.2 ± 0.41 in the PeSeNPs group. Our findings showed that both infrared irradiation treatment alone and PeSeNPs injection alone didn't exhibit a remarkable reduction the tumor development, while in the case of combination therapy (PeSeNPs + infrared radiation), there is a maximum remarkable decrease in tumor volume reached 6.2 ± 0.11, which indicates shrinkage and disappearance of tumor. Infrared light plus PeSeNPs provide powerful photothermal medication and hold promise for other biomedical uses.

##### Mortality rate and survival curve

**Figure 9:**
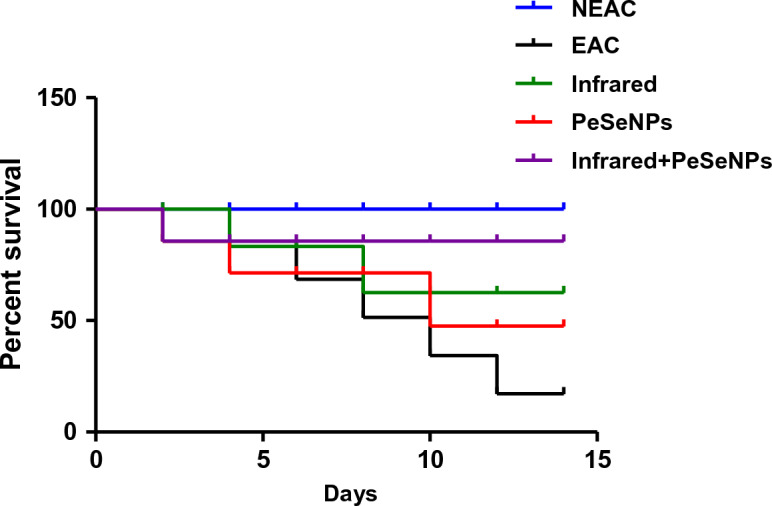
Survival rate of the mice after different treatment.

The rate of mortality is shown in Table 4. There is no percentage mortality of mice that received PeSeNPs and then were irradiated with infrared light. This indicates that the mice's biological system is improving. Combined therapy has been observed for inhibiting the growth of tumor cells. Figure 9 Show the survival curve of mice after numerous treatments.

**Table 4:** Effects of PeSeNPs (225 µg/kg), infrared irradiation, and combined treatment against EAC tumor-bearing mice (n = 10) for each group which represents the total number of mice.

Mortality %	Survivors/total mice	Groups
0	10/10	NEAC
50	5/10	EAC
20	8/10	Infrared
30	7/10	PeSeNPs
0	10/10	Infrared + PeSeNPs

##### Liver enzymes and kidney functions

Table 5. shows an increase in AST and ALT liver enzymes as well as kidney functions, urea, and creatinine in tumor-bearing mice based on the formation of tumor cell growth, which leads to an increase in liver enzymes and kidney functions and therefore there is toxicity in liver and kidney tissue as a result of the tumor development, resulting from excessive stress, which causes interactive oxidative damage, which in turn leads to dysfunction in the liver and kidneys. The observations showed that the group handled with infrared ray and selenium nanoparticles had an improvement in kidney enzymes and kidney functions, which was more effective than infrared ray treatment alone or Nano alone because the mixed treatment had excellent bioavailability due to its low toxicity, strong absorption capacity, and catalytic efficiency. It improves liver and kidney activity and reduces tumor cell metastasis and apoptosis as Se-NPs can modify many genes related to the cell cycle and apoptosis that consequently prevents cancer.

**Table 5:** Liver enzymes and renal function test (n = 10 for each group).

Creatinine (mg/dl)	Urea (mg/dl)	AST (U/L)	ALT (u/l)	Groups
0.93 ± 0.13	33.25 ± 0.24	85 ± 0.21	56 ± 0.33	NEAC
0.98 ± 0.51***	45.44 ± 0.28***	123 ± 0.15***	94 ± 1.15***	EAC
0.88 ± 0.32***	32.16 ± 0.27*	108 ± 2.30***	63 ± 1.31**	Infrared
0.83 ± 0.15**	30.17 ± 1.22*	102 ± 3.12**	60 ± 0.11**	PeSeNPs
0.920 ± 0.22^NS^	32.68 ± 0.74^NS^	83 ± 1.37^NS^	54 ± 0.54^NS^	Infrared PeSeNPs

Each value is expressed as mean ± SE. Non-significant (NS): p > 0.05; Significant (S): *p < 0.05; highly significant (HS): ** p < 0.01; very highly significant (VHS): ***p < 0.001 from NEAC.

### Histopathological examination

#### In tumor tissue

**Figure 10:**
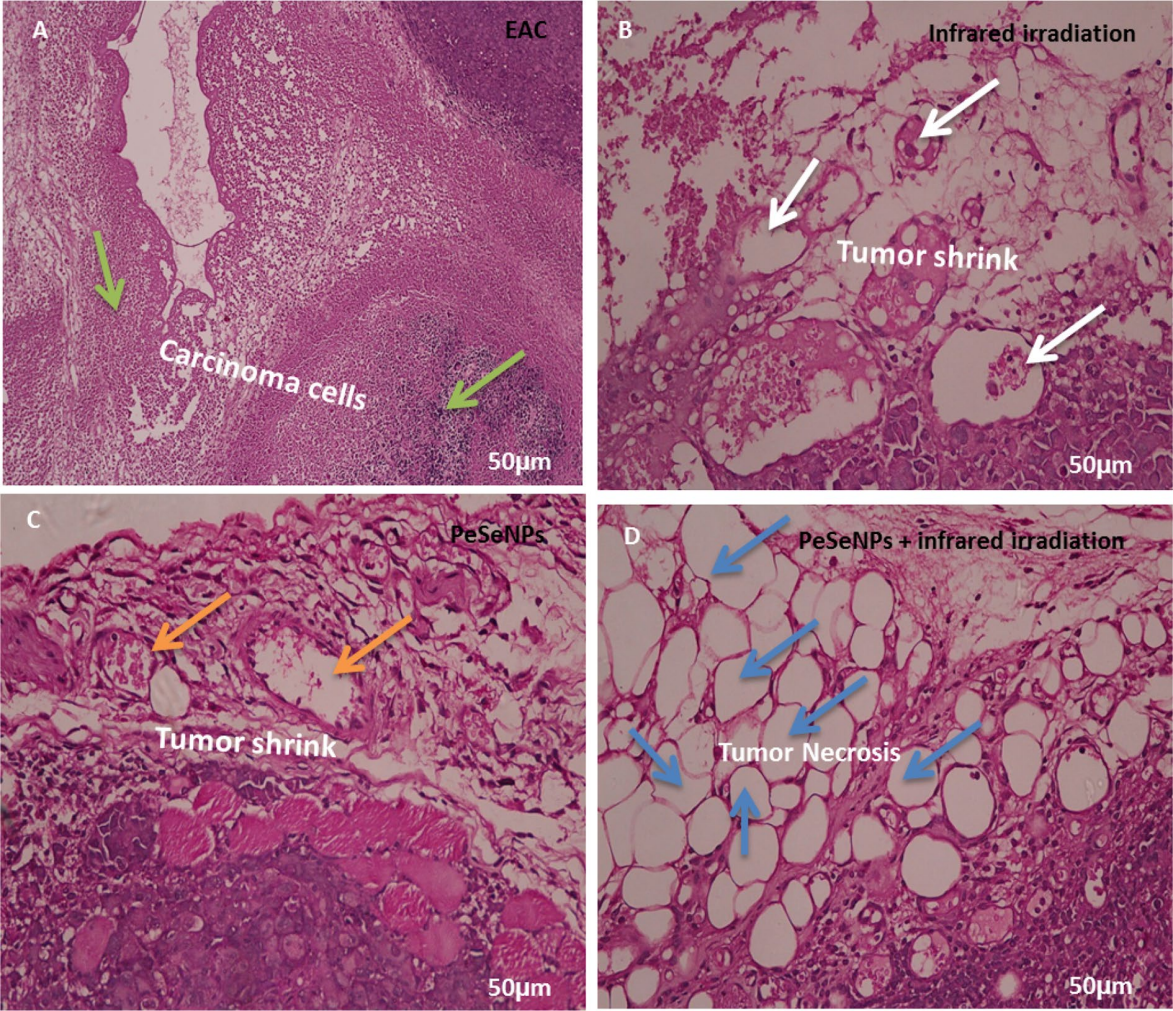
Histological analysis of tumor stained with hematoxylin and eosin (Scale bare: 50 µm). (**A**) Non treated tumor which shows inflammatory cells (green arrow) (**B**) Tumor irradiated with infrared which shows shrink of carcinoma cells (white circle) (**C**) injected tumor with Se-NPs which shows the beginning of cell necrosis (orange arrow circle) (**D**) Tumor injected with Se-NPs then exposed to infrared radiation which showing necrosis cells (blue arrow).

Figure 10 shows the histology of cancer cells stained with hematoxylin and eosin (H&E) (Scale bar: 50 µm). Figure 10A shows tumors without treatment that have inflammatory cells (green arrow). The tumor was irradiated with infrared light only (white arrow, Fig. 10B) and the tumor was also injected with PeSeNPs (orange arrow, Fig. 10C). The two groups showed the beginning of tumor cells shrinkage, but tumors injected with PeSeNPs and then irradiated with infrared light completed necrosis of malignant cells in the core area (Blue arrow, Fig. 10D), therefore PeSeNPs and infrared irradiation illustrated maximum effective destruction of EAC tumor tissue in the comparison to the group of the control^77,78^.

#### In liver tissue

**Figure 11:**
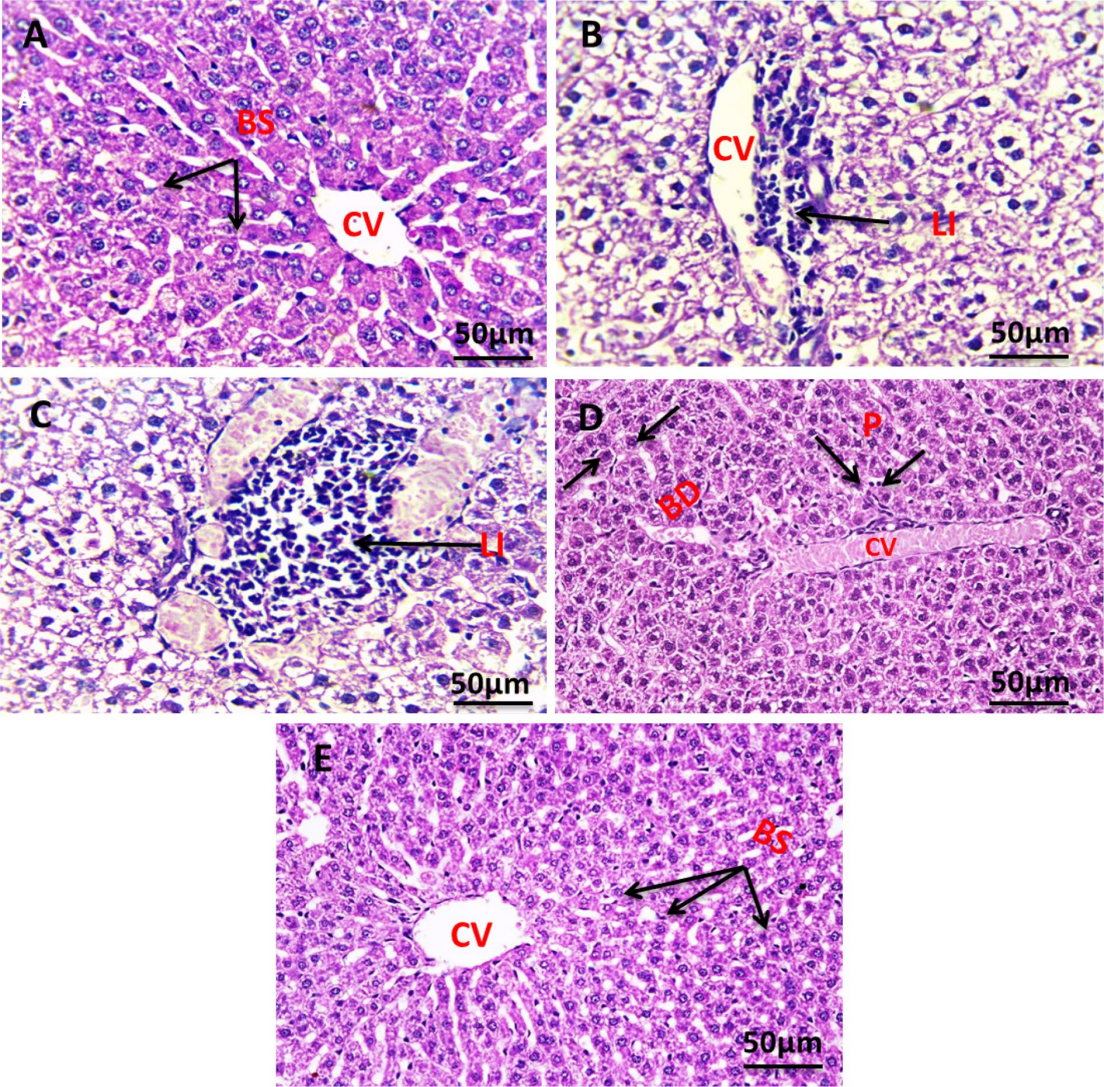
Photomicrograph of liver section of negative control group NEAC showing normal architecture of the liver, central vein (CV), and blood sinusoids (BS) (H. & E., 400X) (**A**). The liver section of the group with untreated tumor showed degenerated hepatocytes with congested central vein (CV), leucocytes infiltration (LI) (H. & E., 400X) (**B**). Liver section of group irradiated tumor with infrared which shows leucocytes infiltration (LI) (H. & E., 400X) (**C**). Liver section of group injected tumor with SeNPs which showed bile ductules (BD) and vacuolated hepatocytes with pyknotic (P) nuclei congested central vein (CV) (H. & E., 400X) (**D**). The liver section of the group injected tumor with SeNPs then irradiated by infrared light showing normal architecture of the liver, central vein (CV), and blood sinusoids (BS) (H. & E., 400X) (**E**).

Figure 11 shows the histology of liver cells stained with hematoxylin and eosin (H&E). Figure 11A indicates the histology of the liver section of the negative control group, which shows a normal architecture of the liver, central vein (CV), and blood sinusoids (BS). The liver section of the group with untreated tumors shows degenerated hepatocytes with congested central vein (CV), and leucocytes infiltration (LI) (Fig. 11B). The liver section of the group irradiated the tumor with infrared which showed leucocytes infiltration (LI) (Fig. 11C). The liver section of the group injected the tumor with SeNPs which showed bile ductules (BD) and vacuolated hepatocytes with pyknotic (P) nuclei congested central vein (CV) (Fig. 11D). The liver section of the group injected tumor with SeNPs then irradiated by infrared light showing normal architecture of the liver, central vein (CV), and blood sinusoids (BS) (Fig. 11E).

#### In kidney tissue

**Figure 12:**
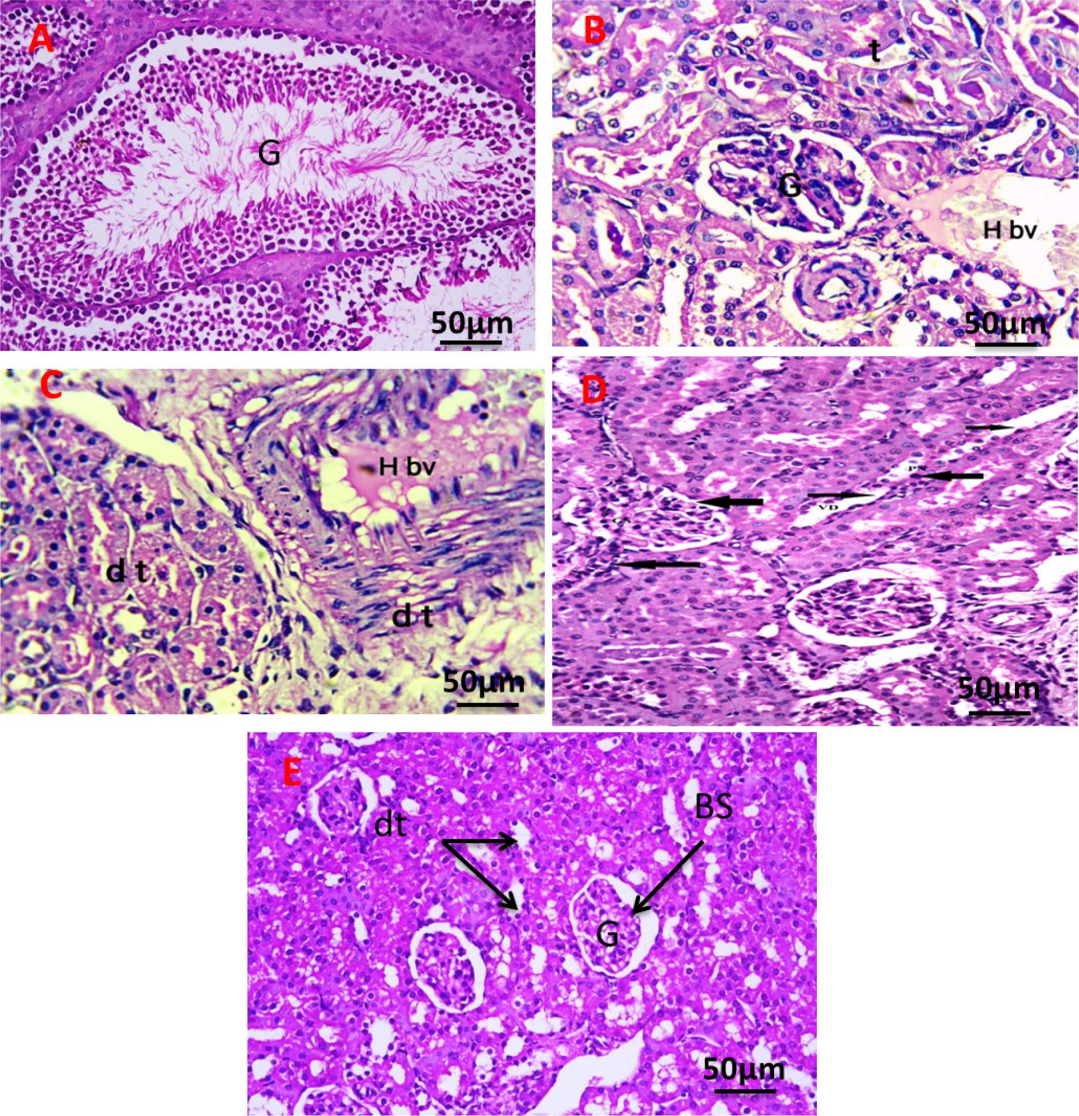
Photomicrograph of Kidney section of negative control group (**A**). The kidney section of group with untreated tumor (**B**), irradiated tumor with infrared (**C**), injected tumor with Se-NPs (**D**), and injected the tumor with Se-NPs then irradiated by infrared light (**E**). All slides were stain by (H & E., 400X).

Figure 12A revealed the histology of the renal section of the negative control group showing normal renal structure of the glomeruli (G), and renal tubules. The kidney section of the group with an untreated tumor, which shows a cross-section in the cortex and medulla region of the kidney in the exposed group, shows degenerative glomeruli with massive aggregation of abnormal nuclei around blood vessels with hemorrhage (Fig. 12B). The kidney section of the group was irradiated with infrared, which showed massive congestion of blood vessels with hemorrhage and vacuolar degeneration in renal tubules with an abnormal nucleus (Fig. 12C). The kidney section of the group injected the tumor with PeSeNPs which showed marked glomerulus sclerosis (GS), pycnotic nuclei (PN), vascular dilation (VD), and necrosis (Fig. 12D). The kidney section of the group injected the tumor with SeNPs then irradiated by infrared light showing normal glomeruli (G), distal tubules (dt), and Bowman's space (Fig. 12E).

### Liver transmission electron microscopy

**Figure 13:**
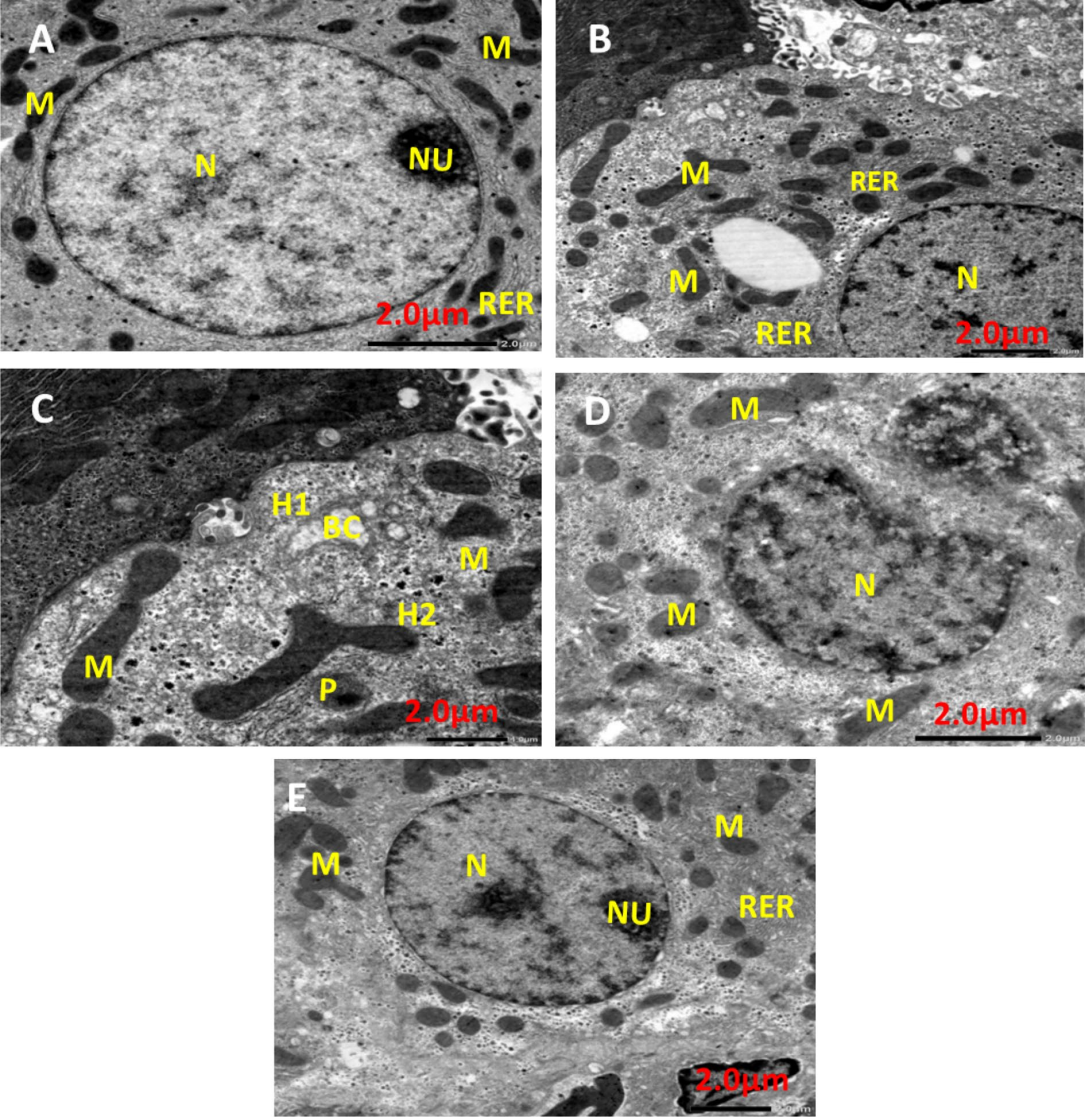
Transmission electron microscope photomicrographs of liver tissue from NEAC, EAC mice treated with, infrared radiation, PeSe-NPs, and PeSe-NPs + infrared radiation.

Figure 13 shows TEM photomicrographs of liver tissue for all experimental groups. The negative control group (Fig. 13A) showed that the cytoplasm contained a round pigmented nucleus (N) having rough endoplasmic reticulum (RER), oval mitochondria (M), and a large nucleolus (Nu). EAC group (Fig. 13B) demonstrated that the nuclei of hepatocytes had clumped, thick chromatin that was arranged erratically into nucleoli (N), and slightly swollen mitochondria (M) and RER dilatation. Infrared group (Fig. 13C) showing Hepatocyte with pyknotic (P) nucleus Illustrating parts of two hepatocytes (H1) and (H2) attached to each other, dilated bile canaliculi (BC) with fragmented microvilli, nucleus (N), rough endoplasmic reticulum (RER), and polymorphic mitochondria (M). PeSeNPs group (Fig. 13D) displays densely granulated, polymorphic mitochondria (M) with aggregated PeSeNP deposition, as well as irregularly shaped nuclei (N). The combined therapy group PeSeNPs + infrared (Fig. 13E**)** showed almost normal hepatocytes structure. The cytoplasm contains a round nucleus (N) with a noticeable nucleolus (Nu), mitochondria (M), and (RER) which remain normal.

## Conclusion

In this work, the newly discovered fungus *Penicillium tardochrysogenum* OR059437 produces PeSeNPs through mycosynthesis. Using several techniques like TEM, UV–vis, zeta potential, EDX, FTIR, and DPPH, PeSeNPs' shape, structure, particle size, and antioxidant activity can all be described. The findings of the TEM showed that the PeSeNPs prepared by mycosynthesis have a consistent sphere shape and an average particle size range of 60.21 to 104.41 nm. The surface of PeSeNPs was analyzed using FTIR to identify functional groups such as carboxyl (C=C), hydroxyl (–OH), phenyl compounds stretching, and C=O of aromatic amide I stretching (proteins and peptides). Through their ability to transport electrons and hydrogen, PeSeNPs demonstrated a significant amount of antioxidant activity against DPPH without the need for stabilizers or capping agents in this study. Also, myco-synthesized PeSeNPs, prove to have strong promising antimicrobial activity towards bacterial and fungal species such as *Penicillium italicum* RCMB 001018 (1) IMI 193019 and *Methicillin-Resistant Staphylococcus aureus* (MRSA) ATCC 4330 and *Porphyromonas gingivalis* RCMB 022001 (1) EMCC 1699. TEM was used to prove the strong antimicrobial of PeSeNPs towards *Penicillium italicum* RCMB 001018 (1), in which, dramatic morphological alterations lead to cell death in *Penicillium italicum* RCMB 001018 (1) IMI 193019 hypha treated by myco-synthesized PeSeNPs. PeSeNPs showed potent therapeutic consequences regarding Ehrlich's ascites carcinoma in vivo alone and in combination with infrared light radiation, So, PeSeNPs represent anticancer agents and a suitable photo thermal option for treating different kinds of cancer cells with lower toxicity and high efficiency to normal cells. The combination therapy showed a very large and significant reduction in tumor volume, the tumor cells showed large necrosis, shrank, and disappeared. There was also improvement in liver ultrastructure, liver enzymes, and histology, as well as renal function, urea, and creatinine.

## Data Availability

All data generated or analyzed during this study are included in this published article.
